# A model study for the progressive disruption of CA1 firing properties during Alzheimer’s disease

**DOI:** 10.1186/1471-2202-12-S1-P49

**Published:** 2011-07-18

**Authors:** Viviana Culmone, Michele Migliore

**Affiliations:** 1 Institute of Biophysics, National Research Council, Palermo, 90146, Italy; 2 Department of Mathematics, University of Palermo, 90123, Italy

## 

Several independent studies show that β-Amyloid (Aβ) peptides accumulation, one of the characteristic hallmark of Alzheimer’s Disease (AD), can affect the normal neuronal activity in different ways causing an increase or a decrease in neuronal membrane excitability. For example, experimental evidence for a negative impact on neuronal membrane in animal models of AD has been obtained in dual patch recordings in rat hippocampal tissue slices, in which Aβ blocked K channels in pyramidal cell dendrites, causing an increase in dendritic membrane excitability. The resulting increased Ca^2+^ influx and excitoxicity may lead to dendritic degeneration. However, further experimental evidence suggests that Aβ may also result in a decrease of cell excitability through its effects on synaptic receptors and other ionic channels. The overall picture is thus somewhat confused, since the interplay of these mechanisms makes difficult to link individual experimental findings with the more general problem of understanding the progression of the disease. This is an important issue, especially for the development of new drugs trying to ameliorate the effect of the disease's progression. Here we first studied the firing properties of a neuron, modeling the different stages of the disease by progressively modifying the intrinsic membrane and synaptic properties taking into account multiple and different experimental findings. We then tested a number of manipulations of channel properties that could compensate for the effects of Aβ The results, obtained under different conditions of channels block and synaptic strength modifications, show the contribution of individual mechanisms to the overall reduction in cell’s excitability, and allow to predict possible therapeutic treatments in terms of pharmacological manipulations of channels kinetic and activation properties. The model is able to show the possible efficacy and collateral effects of different treatments, suggesting how and which mechanism can be targeted by a drug to restore the original firing conditions.

